# Features Associated With Mycoplasma genitalium Infection

**DOI:** 10.7759/cureus.72728

**Published:** 2024-10-30

**Authors:** Johnathan M Sheele, Kara Bragg

**Affiliations:** 1 Emergency Medicine, Mayo Clinic, Jacksonville, USA

**Keywords:** chlamydia, emergency department, gonorrhea, sexually transmitted disease, trichomonas, urethritis

## Abstract

Background

Sexually transmitted infections (STIs) are commonly encountered in the emergency department (ED) in the United States. Limited data exist on the prevalence and risk factors for *Mycoplasma genitalium (M*. *genitalium)*, specifically within the ED. We describe the epidemiological and laboratory findings associated with *M. genitalium* infection at the Mayo Clinic, the whole institution, and specifically in the ED.

Methods

We examined Mayo Clinic Enterprise data for encounters occurring between October 29, 2014, and September 23, 2023, in patients 12-120 years of age who had research authorization present and had testing for *gonorrhea, chlamydia, trichomonas*, and *M. genitalium*.

Results

Among 332,619 encounters, *M. genitalium* testing occurred in 1.7% (n=5,572) of encounters, in which a positive *M. genitalium* test occurred in 1.8% (n=103) of those tested. Since 2020, there has been an increasing trend for *M. genitalium* testing in the ED in the United States, yet testing for *M. genitalium* occurred in <1% of those being tested for other STIs in the ED. *M. genitalium* coinfection in the ED with gonorrhea, chlamydia, or trichomonas occurred <1% of the time for all. Factors significant for *M. genitalium* infection were non-White race (OR 2.33 95% CI (1.50-3.37)), age 45-101 years (vs. 25-35 years) (0.16 (0.05-0.42)), age 36-44 (vs. 25-35 years) (0.47 (0.21-0.94)), married/life partner (vs. divorced, separated, widowed, or unknown) (0.27 (0.11-0.72)), being tested in the ED (versus inpatient and outpatient) and infection with *Ureaplasma* species (3.19 (1.10-9.86)); p<0.04 for all.

Conclusion

*M. genitalium* is infrequently tested in the ED, yet those tested in the ED had a high association with a positive infection. We identified other risk factors for *M. genitalium* infection, including non-White race, age, marital status, and infection with *Ureaplasma* species.

## Introduction

*Mycoplasma genitalium (M. genitalium)* is part of the mollicutes class of small parasitic microorganisms that do not have the peptidoglycan-based rigid cell wall [1]. It resides on and in the mucous epithelial cells in the human genital tract [1]. *M. genitalium* lacks a cell wall, which makes certain antibiotics, such as penicillin and beta-lactams, ineffective [2]. *M. genitalium* detection by culture can take six months, but newer nucleic acid amplification tests (NAAT) have recently been approved by the US Food and Drug Administration (FDA) [2].

*M. genitalium* was first isolated in the 1980s in the urethra of males with nongonococcal urethritis [3]. *M. genitalia* is a slow-growing bacteria with an incubation period of 2-35 days but, in some cases, can be over 60 days [4]. Studies have found that *M. genitalium *causes about 15-20% of nongonococcal urethritis, 20-25% of nongonococcal and non-chlamydia urethritis, and 40% of recurrent or persistent urethritis [2]. *M. genitalium* can be more common in rectal samples than urethral samples in men who have sex with men, but it is unclear if *M. genitalium* is associated with proctitis [5]. The clinical effects of asymptomatic *M. genitalium, *which may represent ~70% of male cases, are less well studied [2,4]. *M. genitalium* infection in women is associated with pelvic inflammatory disease (PID), cervicitis, preterm delivery, spontaneous abortion, and infertility but can be asymptomatic in 40-75% [2-4,6-8]. Untreated *M. genitalium* in females can be cleared in months or up to a year without treatment [1]. *M. genitalium* has been found in 10-30% of women with cervicitis and 4-22% of women with PID [2]. The incidence and associated morbidity of *M. genitalium* is poorly understood, and standards for testing and treatment are still evolving [3,9,10]. For instance, the latest US Centers for Disease Control and Prevention (CDC) Sexually Transmitted Infections (STI) guidelines state that for cervicitis, NAAT testing for *M. genitalium* can be considered, whereas testing for gonorrhea, chlamydia, and trichomonas is recommended [2]. The CDC recommends *M. genitalium* testing in men with recurrent or persistent nongonococcal urethritis and women with recurrent cervicitis [5]. Others have recommended *M. genitalium* testing on persistent cervicitis with consideration of testing at the time of a PID diagnosis, but it is not recommended for routine initial testing for vaginal discharge or asymptomatic pregnant women [7,8,11]. European, British, and Australian guidelines suggest *M. genitalium* testing for cervicitis and postcoital bleeding, PID if a current sexual partner is infected with *M. genitalium* and nongonococcal urethritis in men [5]. *M. genitalium* is not a reportable disease, limiting the ability of researchers to track the incidence and prevalence of the disease [2].

Few studies have explored *M. genitalium* in the emergency department (ED), but they have reported significant differences in the reported prevalence of the bacteria [12-15]. One ED study suggested against screening all ED patients for *M. genitalium* who were also undergoing testing for other STIs as it would result in antibiotic overuse [15]. Clear guidelines on the testing and treatment strategies of *M. genitalium* in the ED are lacking. Our study aimed to assess the epidemiological and laboratory findings associated with *M. genitalium* infection institutionally and in the ED.

## Materials and methods

Study design and setting

The Mayo Clinic institutional review board approved this study (#23-009980). The Mayo Clinic data retrieval team extracted patient encounter data from all Mayo Clinic sites, including Florida, Minnesota, Arizona, and Wisconsin occurring between October 29, 2014, and September 23, 2023, who were between 12-120 years of age, had provided institutional research authorization, and the patient had a polymerase chain reaction (PCR) or nucleic acid amplification test (NAAT) for *Neisseria gonorrhea (N. gonorrhea)*, *Chlamydia trichomonas (C. trichomonas), M. genitalium, Trichomonas vaginalis (T. vaginalis)*, or who had a rapid antigen or genital wet prep for T. vaginalis. The following data were extracted from each encounter: patient's demographics, the encounter's first urinalysis results, the encounter's first urine culture results, the encounter's first genital wet prep results, all STI test results, and the documented reason stated for the patient encounter in the electronic medical record. Text-matching searches in Excel (Microsoft Corp) were used to explore the reasons for the encounter visit. We considered a patient infected with *T. vaginalis* if the patient was positive by urine microscopy, genital wet prep, rapid antigen test, PCR, or NAAT. Patients were considered uninfected with *T. vaginalis* if they had a negative result on the rapid antigen test, PCR, NAAT, or genital wet prep.

Statistical analysis

Categorical variables were summarized using frequency and percentage. Univariable analyses were performed using Fisher's exact test, and multivariable analyses were performed using logistic regression. We chose the following variables to include in the regression analysis based on the results of our univariable analysis: *Ureaplasma spp* (positive for U. *parvum, U. urealyticum*, or both, or negative for both/not tested); *N. gonorrhea, C. trichomonas, and T. vaginalis* (positive for at least one of these STIs vs. negative for all STIs (or testing for the infection was not performed); if the encounter STI testing took place in the emergency department/inpatient vs. outpatient; age (<25, 25-35, 36-44, or 45-101 years of age); marital status (divorced, widowed, separated, or unknown, married/life partner, or single), gender (female vs. not female), race (White vs non-White/unknown), and the state where testing took place (Minnesota vs not Minnesota). All tests were 2-sided, and a p-value of <0.05 was considered statistically significant. Statistical analyses were conducted with BlueSky statistics.

## Results

Of the 332,619 patient encounters in our dataset where gonorrhea, chlamydia, and trichomonas testing took place, only 5,572 (1.7%) had concurrent *M. genitalium* testing performed, with only 103 (1.8%) of those being positive. The median age for patients with an *M. genitalium* test result was 40 (IQR 29; range 12-101 years of age). The majority of patients tested were female gender (3,509; 63.0%), White race (4,739; 85.1%), single (2,129; 38.2%), or married/life partner (2,836; 50.9%), had testing performed in Minnesota (5174; 92.9%), and had no urinalysis testing performed (5083; 91.2%). Approximately 94% of STI testing occurred in the Mayo Clinic Rochester, MN, and the health systems/other sites. *M. genitalium* testing took place in the outpatient (n=5,439; 97.6%), emergency department (n=96; 1.7%), and inpatient (n=37; 0.7%) settings. Positivity rates for *M. genitalium* testing that took place in the outpatient setting were 96/5,439 (1.8%), 6/96 (6.3%) for the emergency department, and 1/37 (2.7%) in the inpatient setting. The positivity and overall testing rates for *M. genitalium* testing in all practices, specifically in the ED (Table 1).

**Table 1:** Positivity rates of M. genitalium by location M. genitalium: Mycoplasma genitalium

	Number of positive M. genitalium tests/total number of tests (%) Inpatient, outpatient, and emergency department	Number of positive M. genitalium tests/total number of tests (%) Emergency department only
October 29, 2014, through 2015	6/556 (1.1%)	0/0 (0%)
2016	8/636 (1.3%)	0/0 (0%)
2017	8/718 (1.1%)	0/0 (0%)
2018	7/583 (1.2%)	0/4 (0%)
2019	10/756 (1.3%)	1/12 (8.3%)
2020	8/613 (1.3%)	0/7 (0%)
2021	14/761 (1.8%)	0/12 (0%)
2022	23/527 (4.4%)	1/19 (5.3%)
2023 (until Sept 22, 2023)	19/402 (4.7%)	4/42 (9.5%)

*M. genitalium* was tested for in the ED 80 times between 2020-2023. Among ED encounters that took place between 2020-2023, the rates of a positive test for gonorrhea were 4.5%, 8.7% for chlamydia, and 4.6% for trichomonas (Table 2). The rates of *M. genitalium* testing were 0.6% for those receiving testing for gonorrhea and chlamydia and 0.4% for those undergoing testing for trichomonas (Table 2).

**Table 2:** Emergency department encounters where testing for gonorrhea, chlamydia, trichomonas, and M. genitalium took place between 2020-2023 *Positive for any one of the following: nucleic acid amplification test (NAAT), genital wet prep, rapid trichomonas antigen test or urine microscopy vs negative for all the following that were performed: NAAT, genital wet prep, and rapid trichomonas antigen test

	Rate of positive test (%)	Frequency of M. genitalium testing performed
Gonorrhea	489/10,848 (4.5%)	62/10,848 (0.6%)
Chlamydia	937/10,818 (8.7%)	61/10,818 (0.6%)
Trichomonas*	314/6,863 (4.6%)	30/6,863 (0.4%)

The following factors were found to be associated with a positive *M. genitalium* test on univariable analysis: infection with *Ureaplasma spp*., getting tested in the emergency department, age, marital status, non-White race, testing taking place outside of Minnesota, and clear urine (p≤ 0.02 for all) (Table 3).

**Table 3:** Factors associated with M. genitalium infection ^ Reference value, OR: odds ratio; CI: confidence interval; M. genitalium: mycoplasma genitalium; HPF: high power field

	+ M. genitalium (n=103)	-M. genitalium (n=5469)	OR (95% CI)	p-value
Ureaplasma parvum	-	-	4.83 (1.74-13.38)	0.004
Positive^	7	443	-	-
Negative	8	2,445	-	-
Ureaplasma urealyticum	-	-	5.72 (1.59-20.52)	0.02
Positive^	3	121	-	-
Negative	12	2,767	-	-
Ureaplasma spp	-	-	0.15 (0.5-0.43)	< 0.001>
Positive^	6	2,351	-	-
Negative	9	537	-	-
Chlamydia trachomatis	-	-	2.62 (0.90-7.57)	0.08
Positive^	4	42	-	-
Negative	52	1,429	-	-
Neisseria gonorrhoeae	-	-	2.18 (0.28-17.08)	0.39
Positive^	1	12	-	-
Negative	55	1,440	-	-
Trichomonas vaginalis	-	-	NA	NA
Positive^	0	14	-	-
Negative	36	1,175	-	-
Chlamydia trachomatis, Neisseria gonorrhoeae, and Trichomonas vaginalis	-	-	2.38 (.92-6.14)	0.08
Positive for at least one^	5	62	-	-
Negative or not tested for infection	58	1,712	-	-
Encounter type	-	-	3.09 (1.41-6.90)	0.01
Emergency or inpatient^	7	126	-	-
Outpatient	96	5,343	-	-
Age (years)	-	-	NA	< 0.001>
<25^	43	806	-	-
25-35	45	1,371	-	-
36-44	10	1,015	-	-
45-101	5	2,277	-	-
Marital status	-	-	NA	< 0.001>
Divorced, widowed, separated, unknown^	8	599	-	-
Married or life partner	11	2,825	-	-
Single	84	2,045	-	-
Gender	-	-	0.70 (0.47-1.03)	0.08
Female^	56	3,453	-	-
Not female	47	2,016	-	-
Race	-	-	0.32 (0.21-0.48)	< 0.001>
White^	67	4,672	-	-
Non-white or unknown	36	797	-	-
State testing took place	-	-	0.31 (0.19-0.51)	<0.001>
Minnesota^	83	5,091	-	-
Non-Minnesota	20	378	-	-
Urinalysis	-	-	0.64 (.28-1.46)	0.38
Urine data present during encounter^	6	483	-	-
No urine data during encounter	97	4,986	-	-
Urine mucus	-	-	NA	1.0
Present^	0	20	-	-
Not reported or none	6	463	-	-
Urine protein	-	-	0.95 (0.17-5.23)	1.0
Negative (0-14 mg/dL)^	4	304	-	-
Positive (>14 mg/dL)	2	144	-	-
Urine pH	-	-	0.10 (0.01-1.17)	0.08
≤7.0^	1	127	-	-
>7.0	2	26	-	-
Urine urobilinogen	-	-	19.33 (1.35-276.40)	0.10
≥2 mg/dL^	1	3	-	-
Normal or negative or <2 mg/dL	2	116	-	-
Urine white blood cells	-	-	2.91 (0.40-21.07)	0.28
≥10 cells/HPF^	2	68	-	-
Occasional, negative, "0-10" cells/HPF, rare	2	198	-	-
Urine epithelial cells	-	-	0.03 (NA)	0.05
None, rare, 0-3 cells/HPF, 1+^	0	69	-	-
Few, moderate, present, >3 cells/HPF	3	40	-	-
Urine nitrite	-	-	0.79 (NA)	1.0
Negative^	3	130	-	-
Positive	0	3	-	-
Urine leukocyte esterase	-	-	0.17 (0.01-1.93)	0.17
Negative^	1	89	-	-
Positive	2	30	-	-
Urine red blood cells	-	-	.56 (NA)	1.0
None, rare, occasional, or 0-10 cells/HPF^	2	211	-	-
Small, moderate, large, or >10 cells/HPF	0	35	-	-
Urine glucose	-	-	3.58 (.65-19.79)	0.20
Negative or <2 mg/dL^	4	154	-	-
Positive	2	276	-	-
Urine clarity	-	-	0.001 (NA)	0.01
Clear^	0	110	-	-
Cloudy or bloody	3	28	-	-
Urine bacteria	-	-	2.28 (0.41-12.62)	0.30
Present^	2	87	-	-
None reported	4	396	-	-
Urine culture	-	-	5.62 (0.75-42.11)	0.12
≥10,000 CFU/mL ^	2	21	-	-
<10,000 CFU/mL	-	118	-	-

On regression analysis, the following were significantly associated with *M. genitalium* infection: *Ureaplasma spp*, age 25-35 years compared with being older than 36 years, being divorced, widowed, separated or unknown marital status compared to being married or having a life partner, non-female gender, and non-White race (p≤0.04 for all) (Table 4). 

**Table 4:** Multivariable regression using infection with M. genitalium infection as the dependent variable M. genitalium: Mycoplasma genitalium; OR: odds ratio; CI: confidence interval

Variables	Comparison groups	Reference levels	OR (95% CI)	p-value
Ureaplasma spp	positive	Negative	3.19 (1.10-9.86)	0.03
-	No data	Negative	5.68 (2.61-14.97)	< 0.001>
Gonorrhea, chlamydia, trichomonas	Positive for at least one infection	No testing	1.35 (0.44-3.33)	0.55
-	Tested for at least one of these infections and that were tested were negative	No testing	0.55 (0.36-0.84)	0.006
Encounter	Outpatient	Inpatient	0.97 (0.41-2.60)	0.95
Age (years)	36-44	25-35	0.47 (0.21-0.94)	0.04
-	45-101	25-35	0.16 (0.05-0.42)	< 0.001>
-	<25	25-35	1.45 (0.92-2.29)	0.11
Marital status	Married or life partner	Divorced, widowed, separated, unknown	0.27 (0.11-0.72)	0.007
-	Single	Divorced, widowed, separated, unknown	0.78 (0.36-1.89)	0.55
Gender	Not female	Female	2.84 (1.84-4.37)	< 0.001>
Race	Not white or unknown	White	2.33 (1.50-3.37)	< 0.001>
State	Not Minnesota	Minnesota	1.31 (0.72-2.27)	0.36
Urinalysis data	Urine data present during the encounter	No urine data during the encounter	0.67 (0.24-1.54)	0.38

Among those with an *M. genitalium* test result, the overall rates of positive STI testing were 10.97% (574/5232) for *Ureaplasma spp*., 3.11% (46/1481) for chlamydia, 1.88% (103/5469) for *M. genitalium*, 1.2% (14/1211) for trichomonas, and 0.87% (13/1495) for gonorrhea. Among all patients receiving *M. genitalium* testing in the emergency department (all years), the rates of positive STI testing were 7/68 (10.29%) for chlamydia, 2/28 (7.14%) for *Ureaplasma spp*, 6/96 (6.25%) for *M. genitalium*, 4/69 (5.80%) for gonorrhea and 1/33 (3.03%) for trichomonas. The rates of coinfection with *M. genitalium* were 4/1,527 (0.3%) for chlamydia, 1/1,508 (0.07%) for gonorrhea, 0/1,225 (0%) for trichomonas, 7/2,903 (0.2%) for *Ureaplasma parvum* and 3/2,803 (0.1%) for *Ureaplasma urealyticum.*

## Discussion

In contrast to the outpatient practice, testing for *M. genitalium* in the ED has been increasing since the first US FDA test for *M. genitalium* was approved on January 23, 2019 [16]. Despite a trend towards increased ED testing for *M. genitalium*, <1% of ED patient encounters with an STI test also had an *M. genitalium* test result. The limited testing for *M. genitalium* at our institution may represent opportunities for provider education. A national survey suggested that *M. genitalium* prevalence was about 1.7% for persons 14-59 years of age, similar to our study's 1.8% positivity rate [11].

Few studies have been published on *M. genitalium* in the ED [12-15.] One ED study found the prevalence of *M. genitalium* in women with nongynecological concerns to be 14.8% [12]. Among these asymptomatic women, 3/9 of them tested positive for chlamydia and also tested positive for *M. genitalium* [12]. *M. genitalium* was detected in 24% of women in the ED with dysuria, which was slightly higher than other practice locations reporting on the rates of *M. genitalium* in patients with dysuria (5-22%) [17].

Studies have found *M. genitalium* coinfection with other STIs, including men with urethritis and gonorrhea, chlamydia, and both gonorrhea and chlamydia were coinfected with M. genitalium 35%, 14% and 19%, respectively [1,18,19]. We found that *M. genitalium* infection was significantly associated with Ureaplasma. Our literature review did not find a previously reported association between *M. genitalium* and Ureaplasma; however, Ureaplasma has been associated with *Mycoplasma hominis* [20]. *M. genitalium* and Ureaplasma are in the class mollicutes and are known to cause urethritis [21]. Among ED patients undergoing testing for *M. genitalium* infection, Ureasplasma was the second most prevalent STI coinfection behind chlamydia. *U. urealyticum* and *U. parvum *can be common in sexually active adults, with a prevalence of >20% in some populations [11,20,21].

Few patients with *M. genitalium* received a concurrent urinalysis, which limited our ability to assess for infection-related urinary changes. However, we did not observe that *M. genitalium* was significantly associated with leukocyte esterase or ≥10 urine white blood cells/high-powered field, as has been seen with other STIs [22,23]. The mollicutes are not known to cause inflammatory vulvovaginitis but have been investigated for an association with bacterial vaginosis [21,24].

We identified that non-White race, age, and marital status were significantly associated with *M. genitalia* infection. These associations have been shown previously to be associated with other STIs [2,22,23,25-27]. Other known risk factors for *M. genitalium* include lower education, Black race, age greater than 30 years, lower socioeconomic status, more sex partners, recent sexual activity, smoking, and a younger age at the time of the first sexual encounter [1,5,12].

Limitations

Our dataset had limited racial, ethnic, and geographic diversity. Among patients being tested for STIs, only 1.7% received testing for *M. genitalium* and only 1.8% of those tested positive. The few positive test results limited broad epidemiological associations within our cohort. Only six patients testing positive for *M. genitalium* also had a urinalysis performed, so our regression analysis did not include findings on urinalysis. *M. genitalium* coinfection with other STIs was rare and only occurred between 0-0.3% of encounters where multiple STI testing took place.

We did not examine all STIs, which anatomic site the swab was collected from, or which assay was used to diagnose it. We did not have the encounter diagnoses and could not assess the indication for STI testing. Some immunocompromised patients are at higher risk for disseminated Mycoplasma and Ureaplasma infections, which could have influenced who was tested in our dataset [20]. *M. genitalium* testing occurring before a test was FDA-approved could have been part of a research study or special circumstances requiring testing with non-FDA-approved diagnostics.

## Conclusions

In our patient population, *M. genitalium* remains rare, although, over the past few years, there has been increased testing, and those tested have a high positivity rate. *M. genitalium* is associated with Ureaplasma spp, non-female gender, non-white race, and in those 12-25 years of age compared to those >25 years of age. Less than 1% of ED patients undergoing testing for other STIs received testing for *M. genitalium*. As awareness of *M. genitalia* becomes more prevalent in the ED community, testing will likely increase. Increased data may yield additional correlations with gender, race, and age, as well as a higher prevalence of the infection than previously documented in the limited population in this study.

## References

[1] David HM, Geisler WM (2024). Mycoplasma genitalium infection. https://www.uptodate.com/contents/mycoplasma-genitalium-infection?%20%20search=mycoplasma%20genitalium&source=search_result.....

[2] Workowski KA, Bachmann LH, Chan PA (2021). Sexually transmitted infections treatment guidelines, 2021. MMWR Recomm Rep.

[3] Gnanadurai R, Fifer H (2020). Mycoplasma genitalium: A Review. Microbiology (Reading).

[4] Lee SJ, Choi JB, Bae S (2024). 2023 Korean sexually transmitted infections treatment guidelines for Mycoplasma genitalium by KAUTII. Investig Clin Urol.

[5] Wood GE, Bradshaw CS, Manhart LE (2023). Update in epidemiology and management of Mycoplasma genitalium infections. Infect Dis Clin North Am.

[6] Anagrius C, Loré B, Jensen JS (2005). Mycoplasma genitalium: Prevalence, clinical significance, and transmission. Sex Transm Infect.

[7] Latimer RL, Vodstrcil LA, Plummer EL (2022). The clinical indications for testing women for Mycoplasma genitalium. Sex Transm Infect.

[8] Frenzer C, Egli-Gany D, Vallely LM, Vallely AJ, Low N (2022). Adverse pregnancy and perinatal outcomes associated with Mycoplasma genitalium: Systematic review and meta-analysis. Sex Transm Infect.

[9] Peel J, Aung E, Bond S, Bradshaw C (2020). Recent advances in understanding and combatting Mycoplasma genitalium. Fac Rev.

[10] Manhart LE, Geisler WM, Bradshaw CS, Jensen JS, Martin DH (2022). Weighing potential benefits and harms of Mycoplasma genitalium testing and treatment approaches. Emerg Infect Dis.

[11] Hufstetler K, Llata E, Miele K, Quilter LA (2024). Clinical updates in sexually transmitted infections, 2024. J Womens Health (Larchmt).

[12] Gragg SD, Gupta KA, Olson KM, Van Der Pol B, Xiao L, Waites KB, Geisler WM (2021). Mycoplasma genitalium infection in young women without urogenital symptoms presenting to a community-based emergency department in Birmingham, Alabama. Sex Transm Dis.

[13] Bayette J, Jreige R, Marchandin H (2013). Prevalence of Chlamydia trachomatis, Neisseria gonorrhoeae and Mycoplasma genitalium infections in the emergency department [Article in French]. Pathol Biol (Paris.

[14] Olson E, Gupta K, Van Der Pol B, Galbraith JW, Geisler WM (2021). Mycoplasma genitalium infection in women reporting dysuria: A pilot study and review of the literature. Int J STD AIDS.

[15] Johnson E, Tieman M, Dumkow K, Pavich E (2024). Comparison of two testing strategies for Mycoplasma genitalium in emergency department patients across a statewide health system. Am J Emerg Med.

[16] (2024). FDA permits marketing of first test to aid in the diagnosis of a sexually- transmitted infection known as. Mycoplasma genitalium. https://www.fda.gov/news-events/press-announcements/fda-permits-marketing-first-test-aid-diagnosis-sexually-transmitted-infection-known-mycoplasma#:~:text=The%20Aptima%20Mycoplasma%20genitalium%20Assay,a%20doctor's%20office%20or%20clinic..

[17] Wilbanks MD, Galbraith JW, Geisler WM (2014). Dysuria in the emergency department: Missed diagnosis of Chlamydia trachomatis. West J Emerg Med.

[18] Tosh AK, Van Der Pol B, Fortenberry JD, Williams JA, Katz BP, Batteiger BE, Orr DP (2007). Mycoplasma genitalium among adolescent women and their partners. J Adolesc Health.

[19] Huppert JS, Mortensen JE, Reed JL, Kahn JA, Rich KD, Hobbs MM (2008). Mycoplasma genitalium detected by transcription-mediated amplification is associated with Chlamydia trachomatis in adolescent women. Sex Transm Dis.

[20] Waites KB (2022). Ureaplasma Infection. Medscape.

[21] Waites KB, Ambalavanan N (2024). Mycoplasma hominis and Ureaplasma infections. UpToDate.

[22] Sheele JM, Niforatos JD, Elkins JM, Campos SC, Thompson CL (2022). Prediction model for gonorrhea, chlamydia, and trichomoniasis in the emergency department. Am J Emerg Med.

[23] Sheele JM, Bragg KJ, Bragg B, Campos SC, Elkins JM, Niforatos JD, Thompson CL (2022). Descriptive epidemiology of women in the emergency department with gonorrhea and Chlamydial infection in the United States. Adv Emerg Nurs J.

[24] Rumyantseva T, Khayrullina G, Guschin A, Donders G (2019). Prevalence of Ureaplasma spp. and Mycoplasma hominis in healthy women and patients with flora alterations. Diagn Microbiol Infect Dis.

[25] Fox HT, Sheele JM (2021). Association of marital status in the testing and treatment of sexually transmitted infections in the emergency department. Cureus.

[26] Elkins JM, Cantillo-Campos S, Thompson C (2020). Descriptive evaluation of male emergency department patients in the United States with gonorrhea and chlamydia. Cureus.

[27] Korich F, Reddy NG, Trent M (2020). Mycoplasma genitalium and Trichomonas vaginalis: Addressing disparities and promoting public health control of two emerging sexually transmitted infections. Curr Opin Pediatr.

